# Transversus Abdominis Plane Block vs. Wound Infiltration for the Reduction of Postoperative Patient-Controlled Analgesia Requirements Following Laparoscopic Hemicolectomy: A Retrospective Case-Control Study

**DOI:** 10.7759/cureus.81707

**Published:** 2025-04-04

**Authors:** Alfie Wright, Thomas Leahy, Charki Chun, Hannah M Delmas, Sam Miller, Pooja Shah

**Affiliations:** 1 Anaesthetics, Southend University Hospital, Southend, GBR; 2 Data Science, Artanis AI, London, GBR; 3 Anaesthetics, Barking, Havering and Redbridge University Hospitals NHS Trust, London, GBR

**Keywords:** laparascopic hemicolectomy, laparoscopy, opioid-sparing analgesia, perioperative analgesia, transversus abdominis plane block (tap block)

## Abstract

Introduction

Transversus abdominis plane (TAP) blocks and wound infiltration are commonly used regional analgesic techniques in laparoscopic colorectal surgery. However, their comparative efficacy remains uncertain. This study aimed to evaluate whether TAP blocks reduce postoperative morphine consumption and patient-controlled analgesia (PCA) duration compared to wound infiltration following laparoscopic hemicolectomy.

Methods

We conducted a retrospective case-control study comparing postoperative opioid requirements and PCA duration in patients who received TAP blocks versus wound infiltration for laparoscopic hemicolectomy. Landmark vs ultrasound-guided TAP block techniques were also compared. The primary outcome was total postoperative morphine consumption via PCA, and the secondary outcome was PCA duration. Data on postoperative adjunct analgesia and patient demographics were also collected.

Results

Comparing TAP blocks (n=59) and wound infiltration (n=33), no significant difference was found between groups in postoperative morphine consumption via PCA (p=0.111), PCA duration (p=0.092), or average daily morphine requirement via PCA (p=0.452). Comparing ultrasound-guided (n=21) and landmark (n=38) TAP block techniques also yielded no significant difference between groups for each of these dependent variables. Variability in opioid use was high, with large standard deviations observed in all groups.

Discussion

TAP blocks did not demonstrate a significant opioid-sparing effect compared to wound infiltration following laparoscopic hemicolectomy. These findings contribute to a growing body of literature with conflicting evidence on the efficacy of TAP blocks. However, TAP blocks may offer benefits beyond postoperative opioid-sparing effects. Further prospective studies incorporating postoperative pain scores and recovery metrics are needed to determine their clinical utility in multimodal analgesia protocols for colorectal surgery.

## Introduction

Significance

Over 13 million laparoscopic procedures are performed globally every year [1]. Options for analgesic techniques for laparoscopy include wound infiltration of local anaesthetic by the surgeon at closure and injection of local anaesthetic into the transversus abdominis plane (TAP) by the anaesthetist after induction of anaesthesia [2]. The TAP is a fascial plane in the anterior abdominal wall lying between the internal oblique and transversus abdominis, which contains terminal somatosensory branches derived from the anterior rami of the lower thoracic and upper lumbar spinal nerves [2]. TAP blocks can consequently potentially provide anterior abdominal wall analgesia from T10-L1 [3]. TAP block procedures vary and can be described both in terms of their approach (subcostal, lateral, posterior or oblique subcostal) and technique (landmark, ultrasound-guided or laparoscopic) [4].

Background

Guidelines produced by the Enhanced Recovery After Surgery (ERAS) Society advocate for the use of TAP blocks in laparoscopic colorectal surgery [5]. However, a recent systematic review shows that evidence for the efficacy of a TAP block in reducing pain following laparoscopic colorectal surgery is mixed [6]. When compared with controls, studies have found TAP blocks to be analgesic and opioid-sparing [7], opioid-sparing but not analgesic [8-10], analgesic but not opioid-sparing [11], and neither analgesic nor opioid-sparing [12,13]. When compared with wound infiltration, several studies did not find TAP blocks to provide superior analgesia [14-16].

Rationale

The studies mentioned above included patients undergoing a variety of laparoscopic colorectal procedures. There is a paucity of published research investigating TAP block efficacy for specific surgeries; for instance, laparoscopic hemicolectomy. A better understanding of the efficacy of different analgesic modalities for specific surgeries could allow for the optimisation of perioperative analgesia, improving the patient’s experience and expediting recovery.

Aims of the study

Examining elective laparoscopic hemicolectomy patients only, this study sought to investigate whether a TAP block administered by the anaesthetist after induction of general anaesthesia is associated with a reduced total postoperative morphine requirement via patient-controlled analgesia (PCA) or a reduced duration of postoperative PCA requirements as compared with wound infiltration. Whether the TAP block technique employed by the anaesthetist (landmark vs. ultrasound (US)-guided) had an effect on these outcome measures was also investigated.

## Materials and methods

Study protocol and ethical considerations

This was a retrospective case-control study with anonymisation of data on collection. Patients who had withdrawn consent for their anonymised data to be used for research purposes were excluded from the study. Approval of the project was granted by the Health Research Authority (IRAS project ID 339559). None of the authors were involved in the care of any of the patients included in this study.

Data collection and eligibility criteria

Data were collected by a retrospective review of PCA charts, anaesthetic records, and operation notes. Data collection was limited to patients who had undergone a laparoscopic hemicolectomy procedure and were managed postoperatively using PCA. Data from a single centre in the southeast of England were used. At this hospital, between January 2020 and March 2024, all patients managed with a PCA were entered into the centre’s Inpatient Pain Service database. This database was filtered to produce an initial sample of such patients who were subsequently assessed for eligibility.

Exclusion criteria were patients who had withdrawn consent for their anonymised data to be used for research purposes; additional operations carried out during the same general anaesthetic, including conversion to laparotomy; use of regional anaesthesia other than preoperative anaesthetist-administered TAP block or wound infiltration in isolation (e.g. spinal anaesthetic block, laparoscopic TAP block); emergency procedure; unclear documentation as to which regional anaesthesia technique was used; recordkeeping error (wrong patient details entered into pain service’s database, or missing anaesthetic record).

Data were tabulated in Microsoft Excel (Microsoft Corporation, Redmond, WA, US). The following data were collected for each patient: age (years), sex, weight (Kg), mode of regional anaesthesia used (TAP block vs wound infiltration), technique used for the TAP block (landmark vs. US-guided), local anaesthetic agent used for regional anaesthesia (all included patients received levobupivacaine), opioid administered in postoperative PCA (all included patients received morphine), total morphine requirement via PCA (mg), whether auxiliary analgesia was prescribed alongside the postoperative PCA, and duration of PCA use (days). For each patient, the average daily morphine requirement whilst on PCA was calculated by dividing the total morphine requirement by the duration of PCA use (mg.day^-1^).

Statistics

Statistical analysis was carried out using JASP [17]. Groups (TAP block vs wound infiltration, landmark technique vs US-guided technique) were compared using Mann-Whitney U tests. Dependent variables were total morphine requirement via PCA, duration of postoperative PCA requirement, and average daily morphine requirement via PCA. The Shapiro-Wilk test was used to assess for deviation in each group’s distribution from normality. Data were not normally distributed, and therefore, descriptive statistics are provided as medians and interquartile ranges. The equality of variances between groups for each dependent variable was assessed using the Brown-Forsythe test.

## Results

Sampling

**Figure 1:**
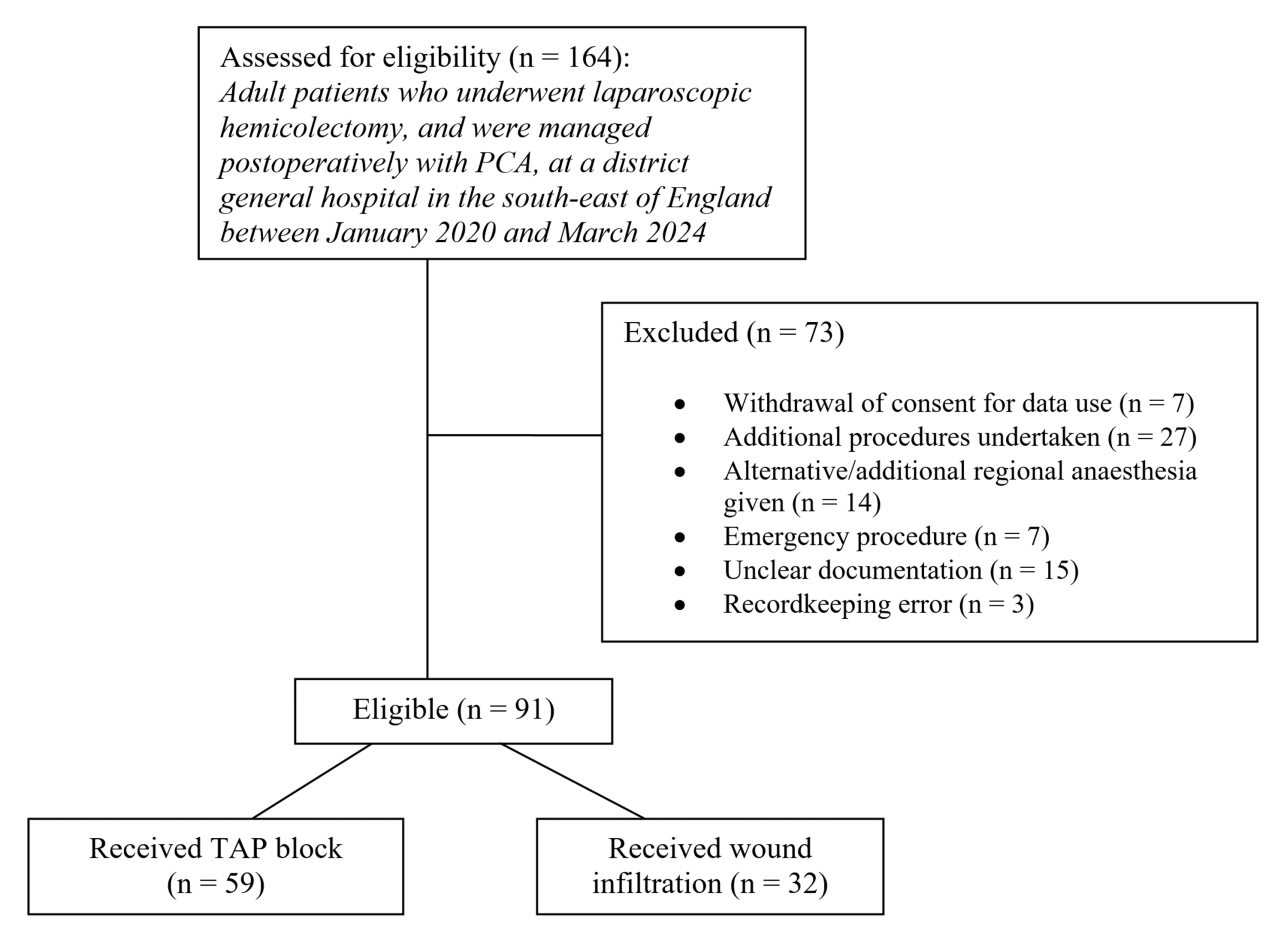
CONSORT diagram illustrating the application of exclusion criteria and the sampling ratio of the dataset CONSORT: Consolidated Standards of Reporting Trials; PCA: patient-controlled analgesia; TAP: transversus abdominis plane

Filtration of the Inpatient Pain Service database produced an initial sample of 164 patients who were subsequently assessed for eligibility. All of these patients underwent a laparoscopic hemicolectomy between January 2020 and March 2024, and all were managed postoperatively with PCA. Following the application of the exclusion criteria, a final sample of 91 eligible patients was reached, as per Figure 1.

Demographics and descriptive statistics

Of the 91 patients deemed eligible for analysis, 45 were male and 46 were female. 59 received a TAP block, and 32 received wound infiltration. Of the patients who received a TAP block, 38 were carried out using the landmark technique and 21 using US guidance. All TAP blocks were performed by experienced anaesthetists. For both TAP blocks and wound infiltration, levobupivacaine was used as the local anaesthetic agent for all patients. All patients received morphine in their PCA cassettes. All patients received regular paracetamol auxiliary to their PCA. Further descriptive statistics are presented in Table 1 and Table 2.

**Table 1:** Descriptive statistics - TAP block vs wound infiltration Descriptive statistics presented as medians and interquartile ranges; 58 patients received a TAP block, and 32 received wound infiltration. TAP: transversus abdominis plane; PCA: patient-controlled analgesia

	Mode of regional anaesthesia	
	TAP block	Wound infiltration
Age (years)	77.00 (15.50)	75.00 (14.00)
Weight (Kg)	74.10 (21.33)	74.50 (25.00)
Total morphine use via PCA (mg)	27.00 (50.50)	44.00(82.50)
Duration of postoperative PCA requirement (days)	2.50 (1.00)	3.00 (2.00)
Average daily morphine requirement via PCA (mg/day)	13.50 (15.17)	20.73 (24.99)

**Table 2:** Descriptive statistics - landmark vs. ultrasound-guided TAP block Descriptive presented as medians and interquartile ranges; 37 TAP blocks were carried out using the landmark technique and 21 were carried out using ultrasound-guidance. TAP: transversus abdominis plane; PCA: patient-controlled analgesia

	TAP block technique	
	Landmark	Ultrasound-Guided
Age (years)	77.00 (16.75)	78.00(12.00)
Weight (Kg)	76.40 (17.75)	75.90 (25.25)
Total morphine use via PCA (mg)	27.50 (47.25)	21.00 (57.00)
Duration of postoperative PCA requirement (days)	2.00 (1.00)	2.00 (1.00)
Average daily morphine requirement via PCA (mg/day)	13.75 (13.53)	10.50 (17.17)

Comparing mode of regional analgesia: TAP block vs. wound Infiltration

Assumption Checks

The Shapiro-Wilk test suggested significant deviation from the normal distribution of data in both the TAP block (p<0.001) and wound infiltration (0.001<p<0.005) groups for all three dependent variables. The Brown-Forsythe test did not suggest significant inequality of variances between the two groups for total morphine requirement via PCA (p=0.127), duration of postoperative PCA requirement (p=0.637) and average daily morphine requirement via PCA (p=0.288).

**Figure 2:**
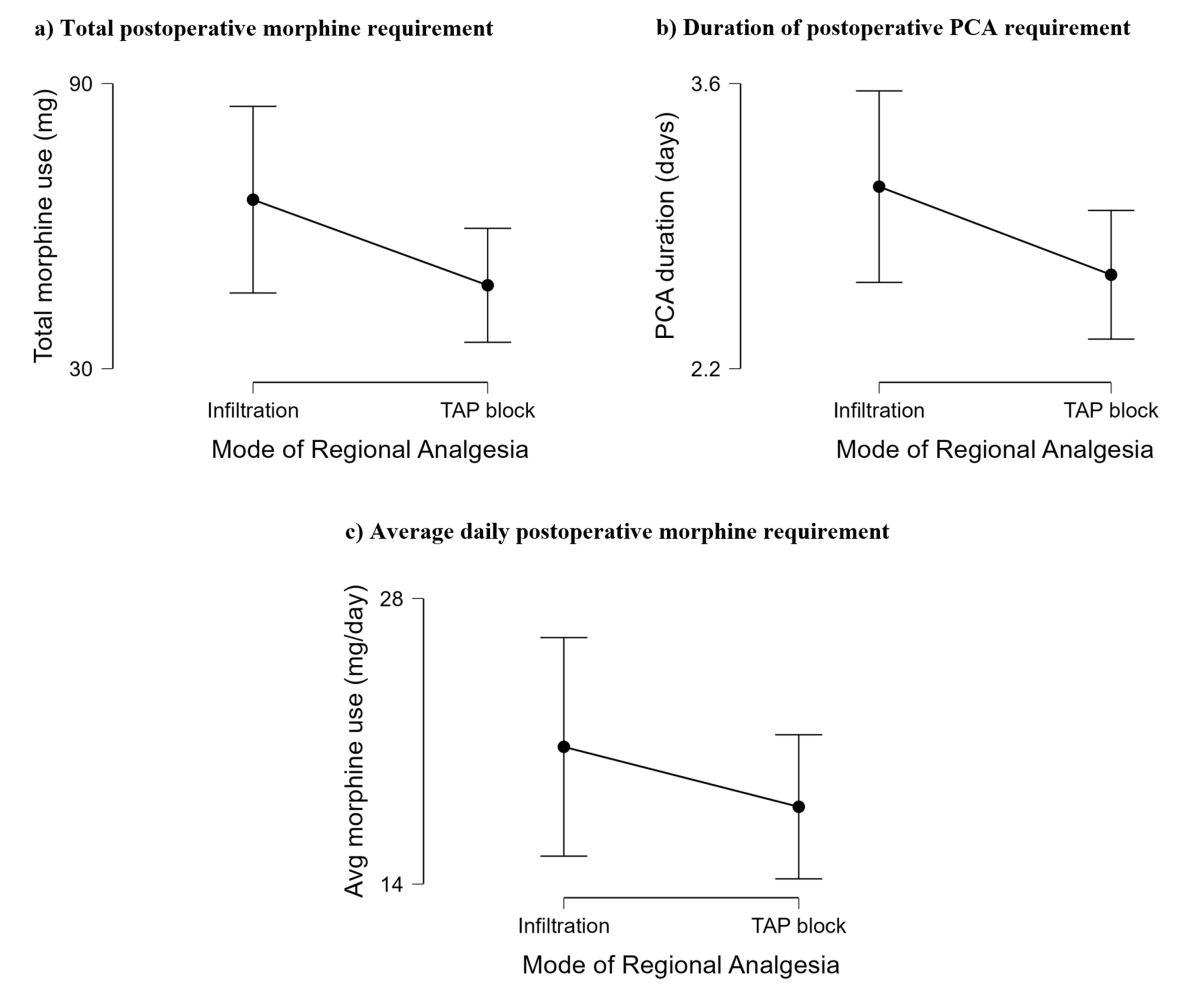
Comparing the effects of the mode of regional analgesia on total postoperative PCA morphine requirement, postoperative PCA duration, and average daily morphine requirement via PCA There was no effect of mode regional analgesia on (a) the total morphine requirement via PCA, (b) the duration of postoperative PCA requirement, or (c) the average daily postoperative morphine requirement via PCA. Data are presented as mean averages ± 95% confidence intervals. TAP: transversus abdominis plane; PCA: patient-controlled analgesia

There was no significant effect of mode of regional block (TAP block vs wound infiltration) on the total morphine requirement via PCA (W=1136, p=0.111), duration of postoperative PCA requirement (W=1136.5, p=0.092), or average daily morphine requirement via PCA (W=1035, p=0.452). These results are presented graphically in Figure 2.

Comparing the TAP block technique: landmark vs ultrasound guidance

Assumption Checks

The Shapiro-Wilk test suggested significant deviation from the normal distribution of data in both the landmark (p<0.001) and US-guided (p=0.001<p<0.008) groups for all three dependent variables. The Brown-Forsythe test did not suggest significant inequality of variances between the two groups for total morphine requirement via PCA (p=0.345), duration of postoperative PCA requirement (p=0.737), and average daily morphine requirement via PCA (p=0.918).

**Figure 3:**
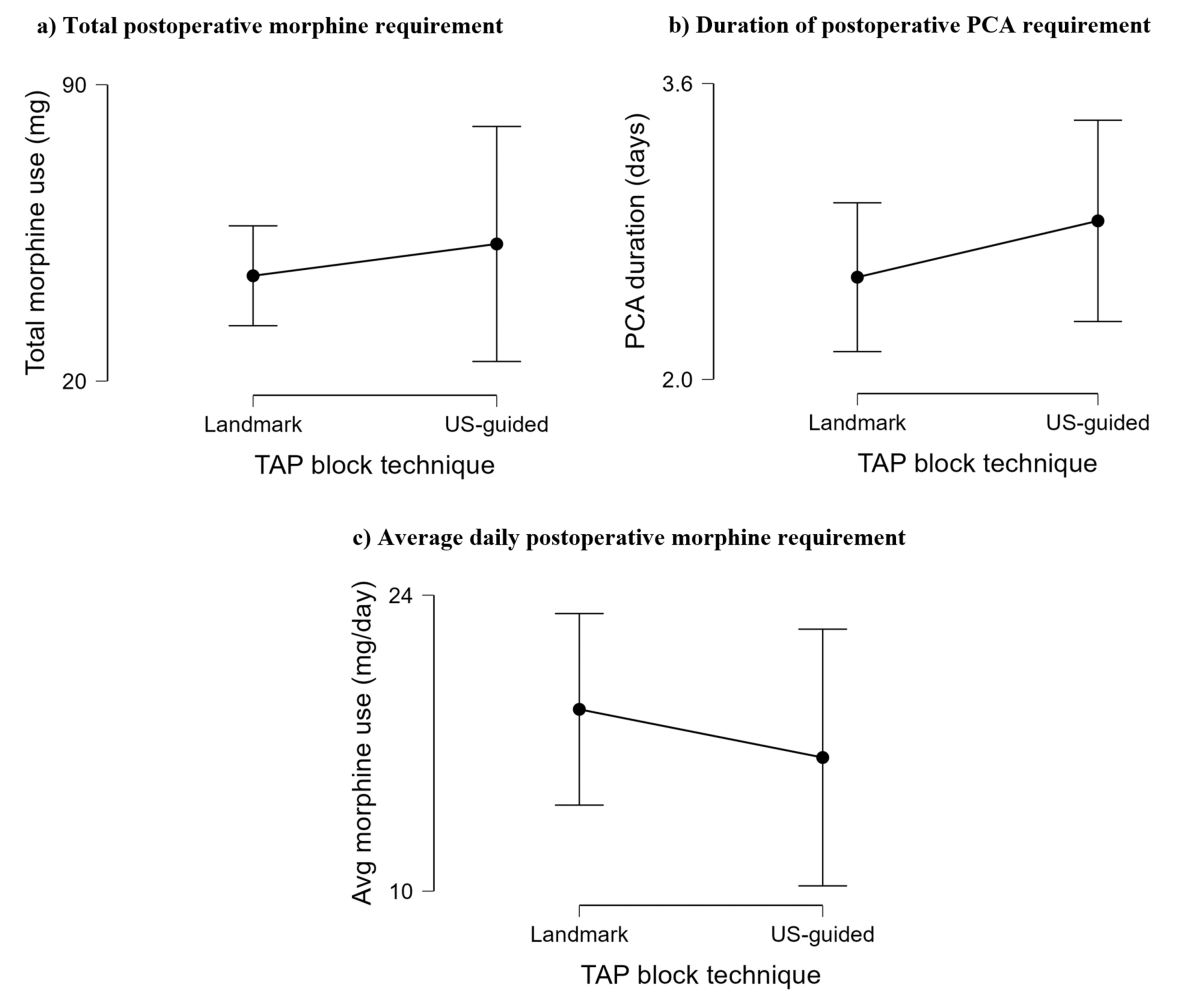
Comparing the effects of the TAP block technique on total postoperative PCA morphine requirement, postoperative PCA duration, and average daily morphine requirement via PCA There was no effect of the TAP block technique on (a) the total morphine requirement via PCA, (b) the duration of postoperative PCA requirement, or (c) the average daily postoperative morphine requirement via PCA. Data are presented as mean averages ± 95% confidence intervals. TAP: transversus abdominis plane; PCA: patient-controlled analgesia; US: ultrasound

There was no significant effect of the TAP block technique (landmark vs US-guided) on total requirement via PCA (W=442, p=0.501), duration of postoperative PCA requirement (W=329.5, p=0.244), or average daily morphine requirement via PCA (W=463.5, p=0.311). These results are presented graphically in Figure 3.

## Discussion

This retrospective case-control study sought to determine whether TAP blocks provide a significant advantage over wound infiltration in reducing postoperative opioid consumption and duration of PCA use in patients undergoing elective laparoscopic hemicolectomy. The results did not demonstrate a significant difference between the two regional analgesia techniques in terms of total morphine requirement, duration of PCA use, or average daily opioid consumption. Additionally, no significant difference was found when comparing landmark and ultrasound (US)-guided TAP blocks for these outcome measures.

Comparison with existing literature

The findings of this study are consistent with previous research suggesting that TAP blocks may not provide superior postoperative analgesia when compared to wound infiltration. Several studies have found that TAP blocks do not significantly reduce postoperative opioid consumption or pain scores compared to wound infiltration in laparoscopic colorectal surgery. While some studies have demonstrated a modest opioid-sparing effect, others have reported no significant difference in analgesic efficacy [7-13]. The mixed results across studies may be attributable to variations in methodology, surgical procedures, and patient populations, as well as the multifactorial nature of postoperative pain. It seems likely that an indwelling TAP catheter, allowing for top-up or continuous analgesia, may be a more efficacious and opioid-sparing approach [18].

Despite the lack of observed clear benefits in postoperative pain control, TAP blocks remain recommended by guidelines, including those from the ERAS Society [5]. However, these recommendations may be based on studies that show benefits in specific subgroups of patients or different surgical procedures. Our findings highlight the need for further research to evaluate the efficacy of TAP blocks in more homogenous surgical populations, as well as to determine whether certain patient groups may derive more benefit than others.

Study limitations

There are several limitations to this study. Although the mode of regional analgesia (TAP block vs wound infiltration) and the technique of TAP block administration (landmark vs US-guided) were accounted for, other potentially influential factors were not controlled for. One key limitation was the inability to standardize intraoperative opioid administration, which may have influenced postoperative opioid consumption, potentially obscuring any difference in analgesic efficacy between TAP blocks and wound infiltration.

Additionally, while all TAP blocks were performed by experienced anaesthetists, the study did not control for the specific approach used (e.g., posterior vs lateral TAP block). Different approaches to TAP block placement may have differing effects on analgesia [4]. For instance, the lateral-to-medial approach has been suggested to offer a better degree of spread of anaesthetic within the plane when compared to lateral and subcostal approaches [19]. Moreover, the volume and concentration of local anaesthetic used were not controlled for beyond ensuring that levobupivacaine was the agent administered in all cases. Future studies should consider these factors to improve the reliability of findings.

A further variable which was not assayed in the present study was postoperative pain scores. These are commonly used as a direct subjective measure of postoperative pain but were not available for our assessment in this study due to the data collection methodology - pain scores were not recorded in the hospital’s Inpatient Pain Service database. We have, therefore, suggested that these scores be incorporated in future iterations of the database to improve the validity of future research.

Another limitation of the study is its retrospective design, which inherently carries risks of selection bias and documentation inaccuracies. While efforts were made to ensure accurate data collection, retrospective studies are limited by the quality and completeness of medical records. Furthermore, the sampling methodology led to a limited sample size, which would be improved upon with a longer data collection window. A prospective, randomized controlled trial would provide a more robust assessment of TAP block efficacy by allowing for stricter standardization of analgesic techniques and perioperative management.

Potential benefits of TAP blocks beyond opioid-sparing effects

Even if TAP blocks do not significantly reduce postoperative opioid consumption compared to wound infiltration, they may still offer other advantages that were not captured in this study. For instance, by providing pre-emptive analgesia, TAP blocks may diminish the impact of surgical stimulation. Elsewhere, it has been suggested that TAP blocks can reduce the need for intraoperative opioids and reduce the incidence of postoperative nausea and vomiting [20].

Additionally, regional anaesthesia techniques, including TAP blocks, have been shown to attenuate inflammatory and neuroendocrine responses to surgery, which may have implications for postoperative recovery and complications. For instance, in a randomised controlled trial of elective laparoscopic cholecystectomy patients, postoperative serum cortisol levels, white blood cell counts, and interleukin assays were lower in patients who had received a TAP block [21]. Importantly, however, this trial compared a TAP block to general anaesthesia (GA) alone, rather than to GA plus wound infiltration, reducing the direct comparability to our study. The ability of TAP blocks to attenuate the surgical stress response is a potential avenue for further investigation.

Another potential advantage of TAP blocks is their impact on postoperative mobilization and recovery. While opioid-sparing effects were not demonstrated in this study, it is possible that patients receiving TAP blocks experience better early postoperative pain control, facilitating earlier ambulation and functional recovery [22]. The effect of regional analgesic techniques on postoperative mobilization and patient-reported outcomes should be considered in future studies.

## Conclusions

The findings of this study suggest that TAP blocks may not provide a clear opioid-sparing benefit over wound infiltration in laparoscopic hemicolectomy patients. Given the additional time, expertise, and resources required to perform TAP blocks, clinicians should consider whether their routine use in this setting is justified. However, the potential benefits beyond opioid reduction, such as haemodynamic stability, attenuation of the surgical stress response, and improved early mobilization, should not be overlooked.

Further research is needed to clarify the role of TAP blocks in perioperative analgesia. Future studies should aim to control for intraoperative opioid use, standardise TAP block approaches and local anaesthetic volumes, and assess additional outcomes such as pain scores, functional recovery, and patient satisfaction. A well-designed prospective trial comparing TAP blocks with wound infiltration in a standardized perioperative setting would provide more definitive evidence to guide clinical practice.
